# The component formula of *Salvia miltiorrhiza* and *Panax* ginseng induces apoptosis and inhibits cell invasion and migration through targeting PTEN in lung cancer cells

**DOI:** 10.18632/oncotarget.21354

**Published:** 2017-09-28

**Authors:** Lei Bi, Xiaojing Yan, Ye Yang, Lei Qian, Yuan Tian, Jian-Hua Mao, Weiping Chen

**Affiliations:** ^1^ School of Preclinical Medicine, Nanjing University of Chinese Medicine, Nanjing 210023, China; ^2^ Biological Systems and Engineering Division, Lawrence Berkeley National Laboratory, Berkeley, CA 94720, USA; ^3^ Changzhou Affiliated Hospital, Nanjing University of Chinese Medicine, Changzhou 213003, China; ^4^ Jiangsu Collaborative Innovation Center of Traditional Chinese Medicine (TCM) Prevention and Treatment of Tumor, Nanjing University of Chinese Medicine, Nanjing 210023, China

**Keywords:** the component formula of *Salvia miltiorrhiza* and *Panax* ginseng (FMG), orthogonal array design, PTEN phosphorylation, PI3K/AKT signaling pathway

## Abstract

Lung cancer still remains the leading cause of cancer-related death worldwide. It is an urgent need for development of novel therapeutic agents to improve current treatment of this disease. Here we investigate whether the effective component formula of traditional Chinese Medicine could serve as new potential therapeutic drugs to treat lung cancer. We optimize the most effective component formula of *Salvia miltiorrhiza* and *Panax* Ginseng (FMG), which is composed of Salvianolic acid A, 20(S)-Ginsenoside and Ginseng polysaccharide. We discovered that FMG selectively inhibited lung cancer cell proliferation and induced apoptosis but had no any cytotoxic effects on normal lung epithelial BEAS-2B cells. Moreover, FMG inhibited lung cancer cell migration and invasion. Mechanistically, we found that FMG significantly promoted p-PTEN expression and subsequently inhibited PI3K/AKT signaling pathway. The phosphatase activity of PTEN protein was increased after FMG bound to PTEN protein, indicating that PTEN is one of the FMG targeted proteins. In addition, FMG regulated expression of some marker proteins relevant to cell apoptosis, migration and invasion. Collectively, these results provide mechanistic insight into the anti-NSCLC of FMG by enhancing the phosphatase activity of PTEN, and suggest that FMG could be as a potential option for lung cancer treatment.

## INTRODUCTION

Lung cancer, including non-small cell lung cancer (NSCLC), is characterized by a low survival, high metastasis and relapse rate after surgery [1–3]. The lung cancer cell proliferation, invasion and migration are the main factors responsible for NSCLC treatment failure [4–6]. The clinical studies indicate that there are some advantages by using traditional Chinese medicine (TCM) to treat lung cancer. TCM can improve symptoms and the quality of life, and extend lifespan of lung cancer patients as well [7]. Therefore, in recent years, the component formula of TCM provides a new prescription module for the treatment of malignant tumors, which composes of clear active components. However, it is acknowledged that a TCM formula is often a complex system, and the effective component(s) and specific target of TCM treatment remain unclear [8].

In traditional Chinese medicine, activating blood circulation to dissipate blood stasis (HuoXueHuaYu) and improving immunity to strengthen healthy (FuZhengPeiBen) are determined to the anticancer therapeutic principle in clinical treatment of lung cancer [9]. According to our previous researches, Radix Salviae Miltiorrhizae et Rhizoma (Danshen) and Radix Ginseng et Rhizoma (Renshen) were chosen for further study, which conformed to this principle and showed remarkable antitumor action [10]. Radix Salviae Miltiorrhizae et Rhizoma (Danshen) is generally considered to be the representative TCM of HuoXueHuayu and its main antitumor action component, Salvianolic acid A (Sal A), has strong inhibitory effects on cell proliferation and migration in A549 cells [10, 11]. And Radix Ginseng et Rhizoma (Renshen) is generally considered to be the representative TCM of FuZhengPeiBen and its major anticancer chemical constituents included Ginsenoside Rh2 and Rg3 and Ginseng polysaccharide (GPS) [12–16]. In this study, we attempt to optimize the most effective component formula of *Salvia miltiorrhiza* and *Panax* Ginseng (FMG), which is composed of Salvianolic acid A (Sal A, 5 μg/mL), 20(S)-Ginsenoside (Rh2, 5μg/mL) and Ginseng polysaccharide (GPS, 10 μg/mL), to investigate whether FMG selectively inhibits lung cancer cell activation but has no cytotoxic effects on normal lung cell BEAS-2B, and to delineate its possible mechanisms through identifying its targeted molecular. Our study demonstrated FMG as a potential option for treating lung cancer.

## RESULTS

### Optimization of the most effective component formula by orthogonal design method

**Figure 1:**
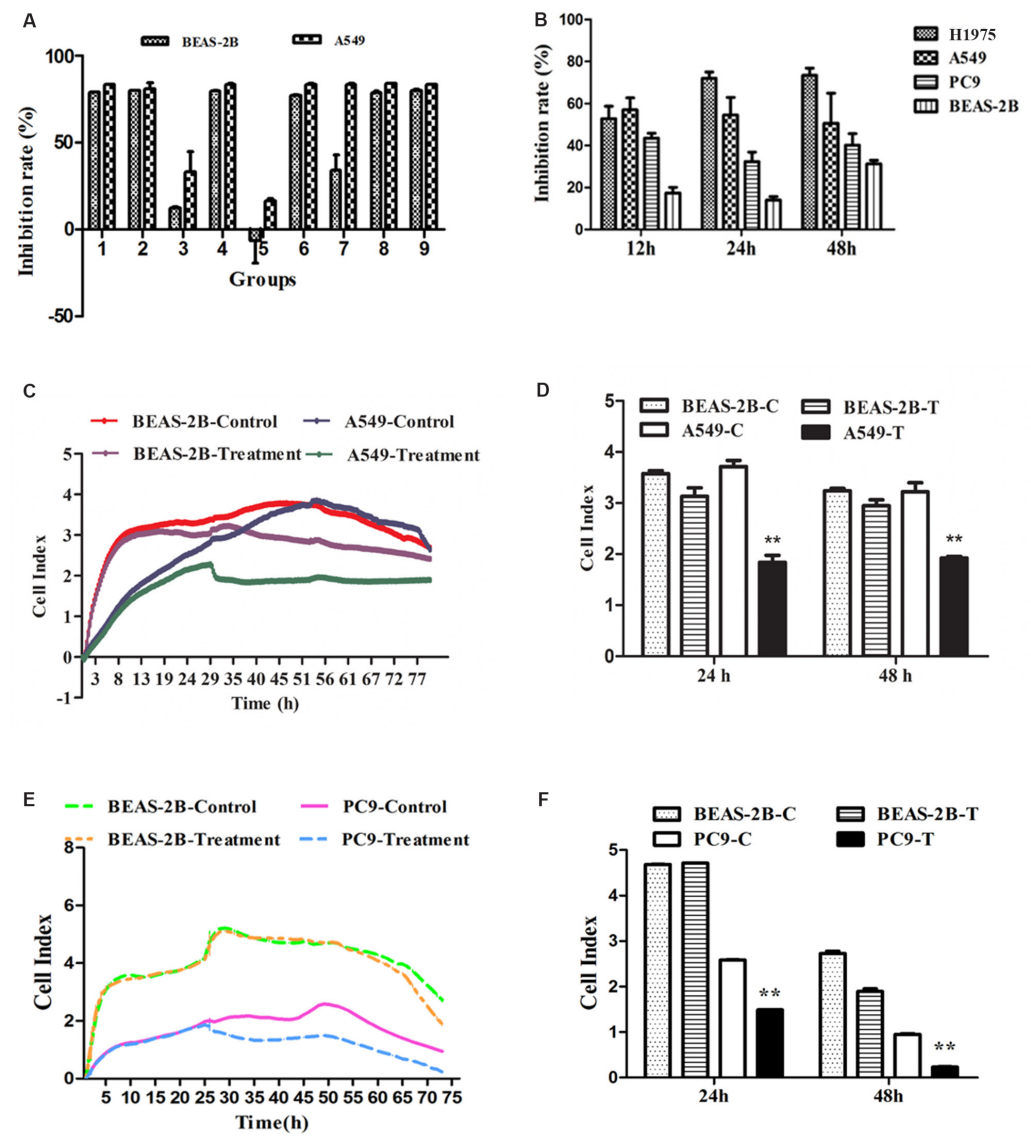
The effects of FMG on lung cancer cell proliferation and viability **(A)** The orthogonal design of L9 (3)^4^ provided 9 groups of different dose combinations. The inhibition ratio of BEAS-2B and A549 cells showed that the optimal combination was Group 5(A2B2D1, FMG). **(B)** Selective inhibitory effects of FMG on lung cancer cells. Cells were treated with FMG for 12 h, 24 h, 48 h, and processed of MTT assay. **(C)** BEAS-2B and A549 cells were seeded in E-plates at density of 1×10^5^ cells/well, cultured for 24 h, and treated with FMG for automated real-time monitoring 48 h in the RTCA-DP instrument. **(D)** The impedance is displayed as a dimensionless parameter termed the “cell index”, which is directly proportional to the total area of tissue culture well that is covered by the cells. Quantification of cell viability on BEAS-2B and A549 cells. Values expressed as mean ± SD from three independent experiments, ^*^P < 0.05, ^**^P < 0.01 *vs.* control group. **(E)** BEAS-2B and PC9 cells were seeded in E-plates at density of 1×10^5^ cells/well, cultured for 24 h, and treated with FMG for automated real-time monitoring 48 h in the RTCA-DP instrument. **(F)** Quantification of cell viability on BEAS-2B and PC9 cells. Values expressed as mean ± SD from three independent experiments, ^*^P < 0.05, ^**^P < 0.01 *vs.* control group.

Anti-lung cancer agents should selectively inhibit the lung cancer cells and be able to protect human normal lung cells, or at least, have no cytotoxicity on normal cells. Hence, firstly, A L9 (3)4 orthogonal array was utilized to optimize the effect of optimal combinations on BEAS-2B and A549 cells. Evaluating the contribution of four factors (antitumor active components) at three dose levels to the growth inhibition of BEAS-2B and A549 cells showed that, the value order was as follows: A1 > A3 > A2, B1 > B3 > B2, C2 > C1 > C3, D3 > D2 > D1 (Figure 1A, Supplementary Tables 1 and 2). The smaller value equated to be stronger inhibitory effect on the lung cancer A549 cells and weaker suppression action on normal lung BEAS-2B cells. Thus, the effect order of factors and levels was as follows: A2 > A3 > A1, B2 > B3 > B1, C3 > C1 > C2, D1 > D2 > D3, and the optimal combination was A2B2C3D1. But owing to the dose of C3 was 0 μg/mL, the optimal combination was changed to A2B2D1, which is composed of Salvianolic acid A (Sal A, 5 μg/mL), 20(S)-Ginsenoside (Rh2, 5μg/mL) and Ginseng polysaccharide (GPS, 10 μg/mL). In order to further uncover the inhibition effect of four factors on BEAS-2B and A549 cells, the analysis of variance showed that the C (Rg3) factor could work severe cytotoxicity on both BEAS-2B and A549 cells (*P* < 0.01) (Supplementary Table 3).

### Effects of FMG on cell proliferation and viability

To verify the effects of optimal combination A2B2D1 (FMG) on lung cancer cells, cell proliferation and viability were determined by MTT assay and the real-time cell analysis (RTCA). Three human cancer cell lines, including A549, H1975, PC9, and human normal lung epithelial cell line BEAS-2B were tested for the proliferation-inhibitory effect of FMG. Compared with the nonmalignant cells, FMG displayed a preferential anti-proliferative activity against lung cancer cells (Figure 1B, Supplementary Table 4). Compared with control group, the viability of A549 and PC9 cells were markedly decreased in FMG-treated group in a time-dependent manner *(P* < 0.01) whereas there was no significant cytotoxic effects on the BEAS-2B cells treated with FMG in 24 h and 48 h (*P* > 0.05) (Figure 1C-1F). These data indicate that the FMG selectively effects on lung cancer cells. Moreover, A549 cells were the most sensitive to FMG. Thus, this lung cancer cell line was chosen as a model to further investigate the antitumor function and mechanisms of FMG.

### Effects of FMG on cell cycle arrest and apoptosis

**Figure 2:**
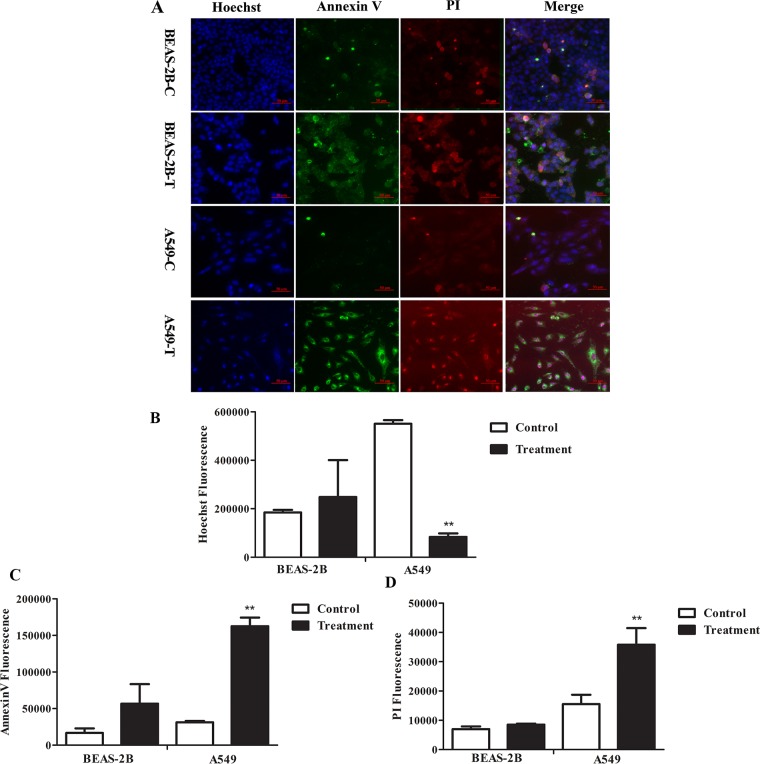
The effects of FMG on cell apoptosis Cells were treated with FMG for 48 h, and then stained with Annexin V-FITC/PI fluorescence dyes. **(A)** Representative images of stained cells were taken by the high-content screening (HCS) AssayScan VTI Reader (×100). Quantification of the Hoechst 33342 **(B)**, Annexin V-FITC **(C)** and PI **(D)** fluorescent intensity by the HCS AssayScan software. Values expressed as mean ± SD from three independent experiments, ^*^P < 0.05, ^**^P < 0.01 vs. control group.

To determine the effects of FMG on cell apoptosis, cells were stained with Hoechst 33342 and Annexin V-FITC/PI and analyzed by high-content screening (HCS) (Figure 2A). Compared with the control group, the Hoechst fluorescence intensity significantly decreased (*P* < 0.01) (Figure 2B), the Annexin V-FITC and PI fluorescence intensity significantly increased (*P* < 0.01) in FMG-treated A549 cells (Figure 2C and 2D). In contrast, in BEAS-2B cells, there was no significant difference in the Hoechst, Annexin V-FITC and PI fluorescence intensity between control and FMG treated groups (*P* > 0.05) (Figure 2B-2D). These results indicate that FMG could selectively induce lung cancer cell apoptosis.

**Figure 3:**
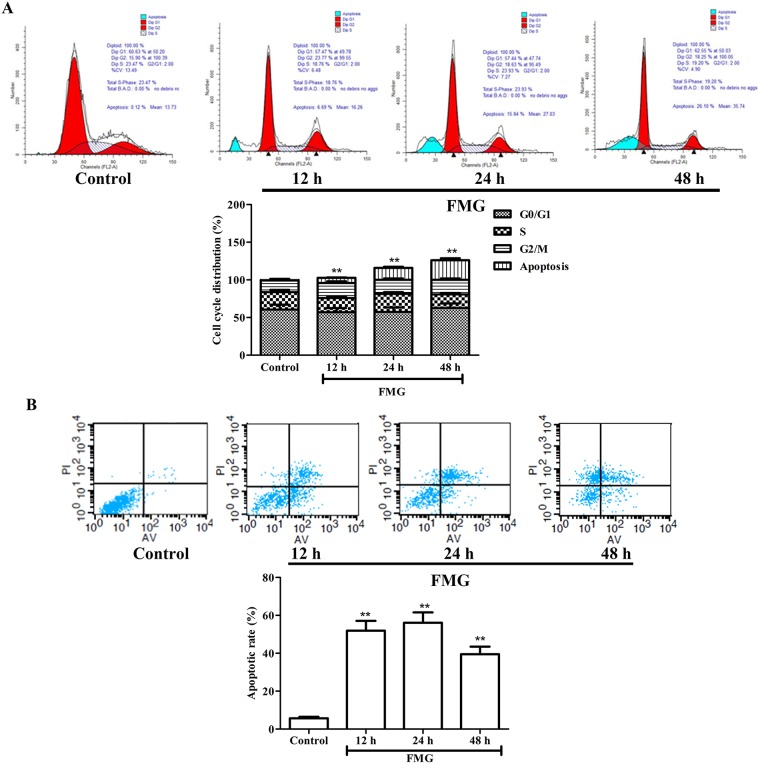
The effects of FMG on A549 cell cycle and apoptosis **(A)** Cells were treated with FMG for 12, 24 and 48 h. The untreated cells were the control group. Cell cycle distribution and cell number percentage in each phase (subG1, G0/G1, S and G2/M) were detected by Flow cytometer. Values expressed as mean ± SD from three independent experiments, ^*^P < 0.05, ^**^P < 0.01 *vs.* control group. **(B)** Annexin V-FITC/PI double staining assay was performed to detect apoptosis of A549 cells using Flow cytometry. Representative images of stained cells were detected. Values expressed as mean ± SD from three independent experiments, ^*^P < 0.05, ^**^P < 0.01 *vs.* control group.

In order to further examine the effects of FMG on cell cycle and apoptosis, cells were further analyzed by Flow cytometry. As shown in Figure 3A, there was no significant difference in A549 cell cycle distribution between the FMG-treated and control group (*P* > 0.05). Flow cytometry analysis showed that the early apoptosis and late apoptotic necrotic cells significantly increased in FMG-treated A549 cells in a time-dependent manner compared with control group (*P* < 0.01) (Figure 3B). These results confirmed that FMG induced cell apoptosis.

### Effects of FMG on cell migration and invasion

**Figure 4:**
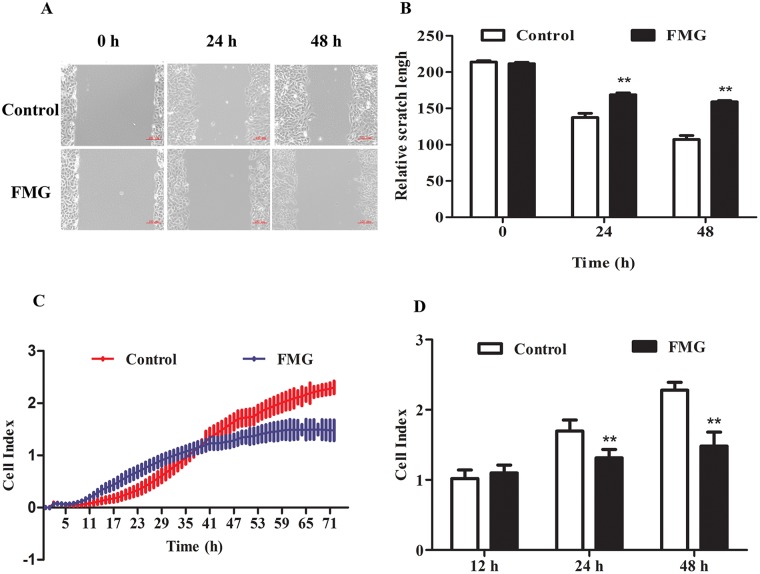
The effects of FMG on cell migration and invasion in A549 cells **(A)** Wound healing assays were used to assess the migration ability of A549 cells after scratches. Cells were exposed to FMG for 24 and 48 h respectively, and then visualized by inverted phase contrast microscope. **(B)** Quantification of the relative scratch length calculated by Imge J software. **(C)** Transwell invasion assayed by Real-time cell analysis (RTCA) were used to monitoring the cell invasion. **(D)** Quantification of invasive cells. Values expressed as mean ± SD from three independent experiments, ^*^P < 0.05, ^**^P < 0.01 *vs.* control group.

To investigate the effects of FMG on the cell migration and invasion, the wound healing and trans-well invasion assay were executed. The wound healing assay revealed that the closure of scratch was significantly slower in the FMG-treated A549 cells than control cells in a time-dependent manner (*P* < 0.01) (Figure 4A and 4B). These data were verified in PC9 and 1795 cells (Supplementary Figure 1). The cell invasion assay further demonstrated that FMG could significantly suppress the A549 cell invasion in 24 and 48 h (*P* < 0.01) (Figure 4C and 4D). Taken together, these findings indicate that FMG inhibits lung cancer cells motility in a time-dependent manner.

### Effects of FMG on the phosphorylation of PTEN

**Figure 5:**
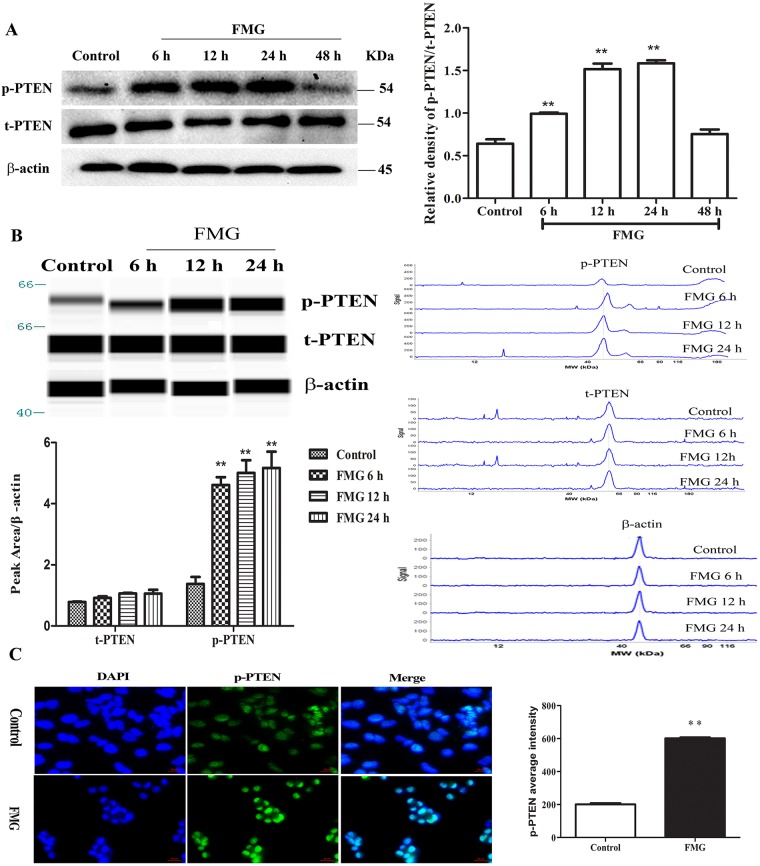
The effects of FMG on the phosphorylation of PTEN in A549 cells **(A)** A549 cells were incubated with FMG for 6, 12, 24 and 48 h, respectively and then the p-PTEN, t-PTEN and β-actin were assessed via western blotting. The untreated cells were the control group. Quantification of p-PTEN/t-PTEN ratio folds of β-actin was calculated by Image J software. Values expressed as mean ± SD from three independent experiments, ^*^P < 0.05, ^**^P < 0.01 vs. control group. **(B)** The phosphorylation of PTEN was detected by simple western analysis. A549 cells were incubated with FMG for 6, 12 and 24 h, respectively and then the p-PTEN and β-actin were assessed via simple western. The pseudo-gel, electropherogram and quantification of simple western were measured using antibody for blotting. Values expressed as mean ± SD from three independent experiments, ^*^P < 0.05, ^**^P < 0.01 vs. control group.**(C)** The phosphorylation of PTEN was stained by immunofluorescence in A549 cells after treatment with FMG for 24 h. Images of stained cells were taken by the high-content screening (HCS) AssayScan VTI Reader (×100). Quantification of p-PTEN average intensity by the HCS AssayScan software, Values expressed as mean ± SD from three independent experiments, ^*^P < 0.05, ^**^P < 0.01 vs. control group.

The phosphatase and tensin homolog gene PTEN, a regulator involved in cell survival, cell cycle, apoptosis and metastasis, is one of the most commonly mutated tumor suppressors in human malignancies [17–19]. To determine which proteins are regulated by FMG, the expression of p-PTEN was measured using western blotting. Strikingly, FMG observably up-regulated the p-PTEN/t-PTEN protein ratio in lung cancer cells compared with control group in a time-dependent manner (*P* < 0.01) (Figure 5A, Supplementary Figure 2). However, the p-PTEN/t-PTEN ratio in cells, which were incubated FMG for 48 h, were not significantly different from in the control. This is probably due to that the cells with high p-PTEN/t-PTEN protein ratio have preceded apoptosis at 48 h.

To further validate the effects of FMG on the phosphorylation of PTEN, we detected the levels of p-PTEN, t-PTEN and β-actin by simple western. Compared with control group, we demonstrated that FMG remarkably increased the expression of p-PTEN in time-dependent manner (*P* < 0.01) (Figure 5B). Furthermore, the expression of p-PTEN was also measured using immunofluorescence and visualized by high-content screening (HCS). As shown in Figure 5C, FMG significantly up-regulated p-PTEN level in A549 cells compared with control group (*P* < 0.01). Taken together, these findings strongly indicate that FMG can up-regulate p-PTEN level in lung cancer cells in a time-dependent manner less than 48 h.

### Effects of FMG on PTEN/PI3K/AKT signaling pathway

**Figure 6:**
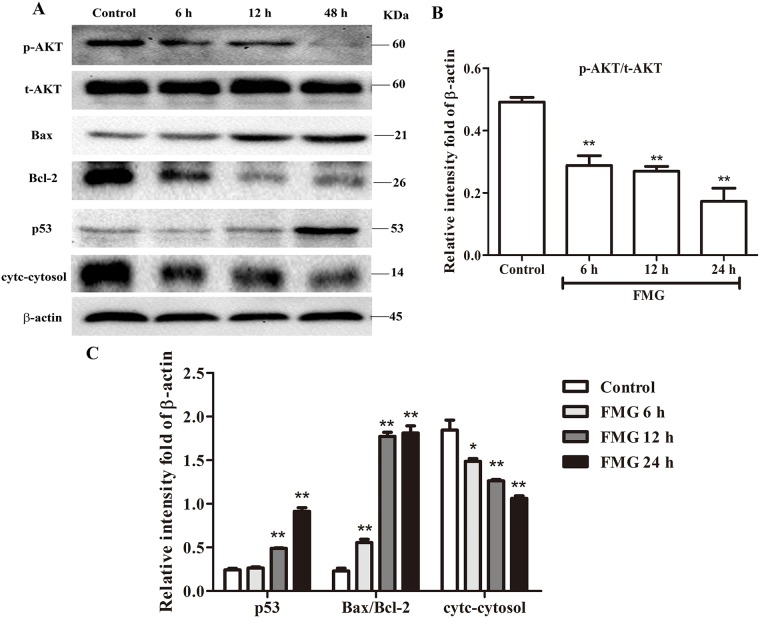
The effects of FMG on PTEN signaling pathway in A549 cells **(A)** A549 cells were in incubated with FMG for 6, 12, 24 h, respectively and then the indicated protein levels of p-AKT, t-AKT, p53, Bax, Bcl-2, cytc-cytosol and β-actin were determined by western blotting. The untreated cells were the control group. **(B)** Quantification of p-AKT/t-AKT ratio, **(C)** p53, Bax/Bcl-2 ratio and cytc-cytosol flod of β-actin were calculated by Image J software. Values expressed as mean ± SD from three independent experiments, ^*^P < 0.05, ^**^P < 0.01 *vs.* control group.

Phosphoinositide 3-kinase (PI3K)/Akt signaling pathway is essential in the regulation of cell survival, apoptosis, migration and invasion and PTEN has been demonstrated to be the essential molecule regulating PI3K/AKT pathways [20, 21]. We next investigated whether the PTEN/PI3K/AKT pathway is responsible for that FMG induced cell apoptosis and suppressed cell migration and invasion. As shown in Figure 6A and 6B, FMG could significantly decrease the protein ratio of p-AKT/t-AKT, suggesting that FMG markedly suppressed PI3K/AKT signaling pathway. To determine which apoptotic proteins are regulated by FMG through PI3K/AKT, the expression of p53, Bax/Bcl-2 and cytc-cytosol were measured using western blotting analysis. We observed that the levels of p53 and Bax/Bcl-2 significantly increased in FMG-treated A549 cells compared with the control group (*P* < 0.01). And the level of cytc-cytosol significantly decreased in FMG group compared with the control group (*P* < 0.01) (Figure 6A and 6C).

### Effects of FMG on cell cytoskeletal rearrangement

**Figure 7:**
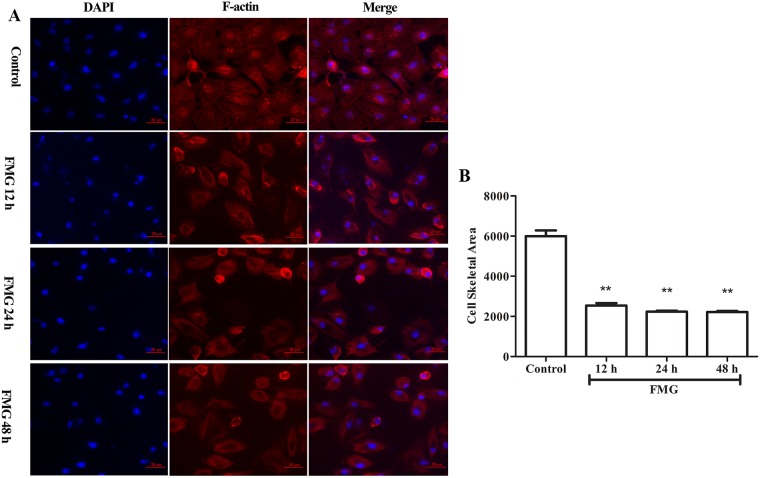
The effects of FMG on cell cytoskeletal rearrangement in A549 cells Cells were treated with FMG for 12, 24, 48 h, respectively, and then stained with DAPI, F-actin fluorescent dyes. **(A)** Images of stained cells were taken by the high-content screening (HCS) AssayScan VTI Reader (×100). **(B)** Quantification of cell skeletal area value was calculated by the HCS AssayScan software. Values expressed as mean ± SD from three independent experiments, ^*^P < 0.05, ^**^P < 0.01 *vs.* control group.

F-actin filaments play important roles in mitosis, cell signaling, and motility, and the cytoskeletal rearrangement is often associated with the metastatic potential [22]. Thus, the cytoskeletal filaments are the targets of anti-cancer drugs. Next, Rhodamine phalloidin was used to evaluate the changes of F-actin filaments. As shown in Figure 7, FMG significantly decreased cell skeletal area of F-actin filaments in time-dependent manner compared with control group (*P* < 0.01).

### FMG directly targets PTEN protein

**Figure 8:**
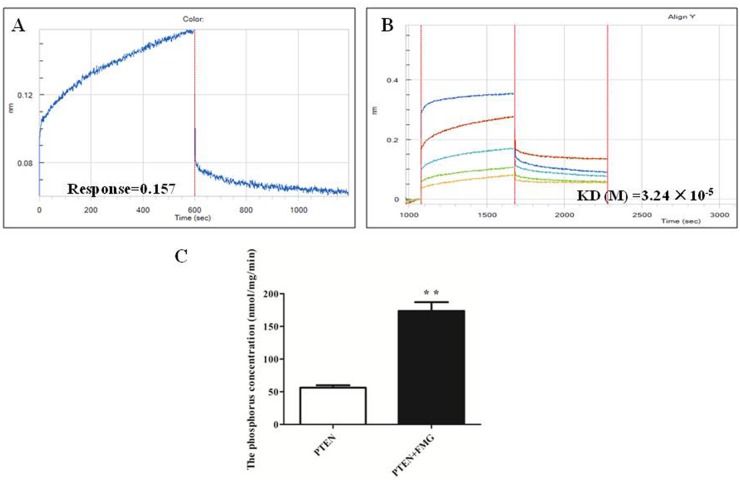
The FMG interaction with PTEN protein was analyzed by Octet RED (ForteBio) **(A)** The Association and dissociation curves and **(B)** affinity of the FMG and PTEN protein were calculated *via* the ForteBio software. **(C)** The phosphatase activity of PTEN protein after interaction of FMG was detected through PTEN phosphatase activity assay kit. Values expressed as mean ± SD from three independent experiments, ^*^P < 0.05, ^**^P < 0.01 *vs.* control group.

To gain further insight into whether PTEN is the molecular target of FMG, the His-tagged PTEN fusion protein was constructed by prokaryotic expression vector, the His-PTEN was purified by the ÄKTA purifier M1 protein chromatography purification system and the kinetics of FMG binding to PTEN protein was then assessed using the Octet RED. As shown in Figure 8A, the kinetic binding curves demonstrated that FMG interacted with PTEN protein and the value of response was 0.157, indicating FMG directly targeted into PTEN protein. Moreover, the kinetic analysis of FMG binding to PTEN protein at different concentrations revealed that the affinity value (KD) of FMG and PTEN protein was 3.24×10^-5^ (Figure 8B, Supplementary Table 5), suggesting that FMG strongly interacted with PTEN protein. Next, we investigated the phosphatase activity of PTEN protein after FMG binding to PTEN protein *in vitro*, and observed that FMG markedly increased the phosphatase activity of PTEN protein (*P* < 0.01) (Figure 8C), which indicated that FMG could promote cell apoptosis and inhibit cell migration and invasion through targeting PTEN protein and increasing the PTEN phosphatase activity.

## DISCUSSION

Lung cancer is the most common malignant tumor in the world and has the highest fatality rate. Even after clinical treatment, five-year survival rate is still low [23–25]. Current clinical evidence has indicated that in comparison to patients with non-small cell lung cancer (NSCLC) receiving chemotherapy, the patients using traditional Chinese medicine (TCM) had longer lifespan and higher quality of life [26]. Thus, the component formula with definite and effective components provided new opportunity to treatment lung cancer. However, the specific mechanism of TCM treatment still remains unclear.

Nowadays, targeted therapy has become the most promising therapy and a welcome asset to the lung cancer therapeutic arena [27, 28]. And an ideal cancer drug specifically targets cancer cells while sparing normal tissues [29]. Therefore, firstly, in present study, the orthogonal design method was used to optimize the most effective formula component of FMG and we found that the optimal combination FMG was A2B2D1 (Sal A 5 μg/mL, Rh 5 μg/mL and GPS 10 μg/mL). Subsequently, we showed that FMG could selectively inhibit lung cancer cell proliferation and induce cell apoptosis whereas FMG have no obvious cytotoxicity on normal lung cell BEAS-2B. This property is critically important for exploiting FMG as a potential anti-lung cancer therapy. This was supported by observations that FMG markedly suppressed lung cancer cell migration and invasion in time course manner. FMG significantly decreased cell skeletal area of F-actin filaments in time-dependent manner. F-actin filaments are the main constituent of microfilament cytoskeleton proteins, which is very important role in cancer cell migration and invasion.

FMG not only selectively inhibited cell proliferation and induced apoptosis in lung cancer cell lines, but also suppressed cell migration and invasion, which put forward the hypothesis that FMG could bind to distinct sites of protein. PTEN (Phosphatase and tension homolog deleted on chromosome 10) originally acts as tumor suppressor, which frequently lost or mutated in various human malignances [9, 30]. PTEN is a 403 amino acid and dual lipid/protein phosphatase which can change second messenger PIP3 (phosphatidylinositol 3,4,5-triphosphate) to PIP2 (phosphatidylinositol 4,5-bisphosphate), and inhibits its downstream PI3K/AKT signaling pathway associated with cell proliferation, apoptosis, migration and invasion [31]. Our data strongly demonstrated that FMG up-regulated p-PTEN expression and markedly suppressed PI3K/AKT signaling pathway in lung cancer cells. Further, our results suggested that the mechanism for FMG induced cell apoptosis and inhibited cell migration and invasion *via* regulation of p53, Bax/Bcl-2, cyto-cytosol and F-actin.

Although we found that up-regulation of PTEN phosphorylation and suppression of PI3K/AKT signaling pathway were required for FMG-induced apoptosis, migration and invasion. In order to further prove that PTEN is the molecular target of FMG, we constructed and expressed the His-tagged PTEN fusion protein by prokaryotic expression vector, and purified the His-PTEN, and then assessed the interaction and kinetics of FMG binding to PTEN protein using the Octet RED. For the first time, we demonstrated that FMG directly targeted into PTEN protein and had strong affinity. And the phosphatase activity of PTEN protein after FMG binding to PTEN protein *in vitro* suggested that FMG significantly promoted the phosphatase activity of PTEN protein. Taken together, the mechanism for FMG inducing cancer cell apoptosis and inhibiting cell migration and invasion may be through targeting PTEN protein and enhancing the PTEN phosphatase activity.

In summary, our studies optimized the FMG, which selectively effected lung cancer cells, induced cell apoptosis and inhibited cell migration and invasion but had no cytotoxic effects on normal lung cell BEAS-2B. Up-regulation of p-PTEN and suppression of PI3K/AKT signaling pathway by FMG resulted in pro-apoptosis action and prevention of cancer cell migration and invasion. Molecular binding evidence indicated that PTEN could be a direct target molecular for FMG within cancer cells. Our results provided mechanistic insights into FMG inhibition of lung cancer cell proliferation, migration and invasion and implicated FMG as anti-lung cancer therapy.

## MATERIALS AND METHODS

### Chemicals and reagents

Salvianolic acid A (Sal A) was provided by Shanghai Yousi Biotechnology Co., Ltd. (Shanghai, China). 20(S)-Ginsenoside (Rh2) and 20(R)-Propanaxadiol (Rg3) were provided by National Institute for control of pharmaceutical and biological products (Beijing, China). Ginseng polysaccharide (GPS) was purchased from Nanjing Zelang Medicine Technology Co., Ltd. (Nanjing, China). Paclitaxel was purchased from Sichuan Taiji Pharmaceutical Co., Ltd. Taiji Group (Chengdu, China). Cisplatin was purchased from Nanjing Pharmaceutical Co., Ltd. (Nanjing, China). Dulbecco's modified Eagle's medium (DMEM), Fetal Bovine Serum (FBS), penicillin and streptomycin were purchased from Gibco (Grand Island, NY, USA). Cell Counting Kit-8 (CCK-8) was purchased from Dojindo Laboratories Science and Technology, Inc., (Kamimashiki gun Kumamoto, Japan). Propidium iodide (PI) and IPTG were purchased from Sigma-Aldrich, Sigma Chemical Co., (St. Louis, MO, USA). Cellomics Cell Motility kit, Cellomics Cytoskeletal Rearrangement kit, Hoechst 33342 staining kit, Alexa Fluor Annxin V and PI double staining kit were purchased from Thermo Fisher Scientific Inc., (Massachusetts, USA). Anti-β-actin, anti-PTEN, anti-p-PTEN, anti-AKT, anti-p-AKT were purchased from Cell signaling Technology Inc., (Boston, MA, USA). Anti-Bax, anti-Bcl-2, anti-p53 were purchased from Abcam Inc., (Cambridge, UK). Anti-cyt-*c* was purchased from Epitmics Inc., (Burlingame, CA, USA). Matrigel matrix was purchased from BD Biosciences (Sanjose, CA, USA). Simon kit was purchased from ProteinSimple Inc., (Santa Clara, CA, USA). DNA polymerase, T4 DNA ligase, BamH I, Hind III were obtained from TaKaRa Bioindustry Inc., (Dalian, China). HisTrap HP was purchased from GE Healthcare Life Sciences (Marlborough, MA, US). PTEN phosphatase activity assay kit was purchased from GenMed Scientifics Inc., (Arlington, MA, USA).

### Cell lines and cell culture

Human normal lung epithelial cell line BEAS-2B and human lung epithelial tumor cell line A549 were purchased from Shanghai Institute of Biochemistry and Cell Biology. NSCLC NCI-H1975 cells were obtained from Shanghai Cell Bank. The PC9 cell line, which was derived from a human adenocarcinoma of lung tissue, was a gift from Dr. Jian-hua Mao (Lawrence Berkeley National Laboratory, USA). Cells were cultured in Dulbecco's modified Eagle's medium (DMEM) and supplemented with 10% heat-inactivated FBS and 100 unit/ml penicillin, and 100 μg /ml streptomycin at 37°C with a humidified atmosphere of 5% CO2.

### Orthogonal design

The orthogonal design of L9(3)^4^ was employed to optimize the most effective component formula of Salviae Miltiorrhizae Radix et Rhizoma and Ginseng Radix et Rhizoma (FMG) on lung cancer cell A549 and normal lung epithelial cell BEAS-2B (Supplementary Table 6). Data were analyzed by orthogonal Design of SPSS 17.0 software.

### CCK-8 assay

Cell viability was assayed by Cell Counting Kit-8 (CCK-8) as described [32, 33]. Briefly, BEAS-2B and A549 cells were seeded into 96-well plates (Costar, Corning Inc., Corning, NY) at density of 1×10^4^ cells/well, cultured for 24 h, and then divided into 9 groups (Supplementary Table 7). After incubation for 24 h, the cell viability was assayed by CCK-8. All experiments were performed at least three times. Data were calculated as the percentage of cell Inhibition Rate (%) = (1- sample solution absorbance value/control absorbance value)× 100%.

### Monitoring cell adherence and proliferation

Cell proliferation was assessed by MTT assay. Briefly, BEAS-2B, A549, PC9 and H1975 cells were seeded in 96-well plates at the density of 4 × 10^4^ cells/mL. After cultured for 24 h, the cells were treated with FMG or vehicle (DMSO, 0.1%) in MEM medium containing 10% (v/v) FBS for 12 h, 24 h and 48 h. Then, MTT (5 mg/mL) was added into the medium following incubation for 4 h at 37 °C. The MTT medium was discarded and the cells were lysed in 150 μl of DMSO each well. The optical density (OD) at 490 nm was measured using a Microplate Reader (Biotek ELx800, Winooski, VT, USA). All experiments were performed at least three times. Data were calculated as the percentage of cell Inhibition Rate (%) = (1- sample solution absorbance value/control absorbance value)× 100%. The blank is the well which contains culture medium but no cells.

To monitor cellular adhesion and growth responses we have exploited the system from Roche Applied Sciences, consisting of microtiter plates (E-plates) with integrated gold microarrays in the bottom of wells for continuous and label-free measurements of cellular status in real-time by the Real-time Cell Analyzer-Dual Purpose (RTCA DP) instrument [34–36]. In our study, cells were seeded in E-plates at density of 1×10^5^ cells/well, cultured for 24 h, and treated with FMG. And then the E-plates were transferred to the RTCA-DP instrument for automated real-time monitoring at standard incubator conditions, quadruplet readout of the parameter “Cell Index” every one hour the following 48 hours.

### High content screening (HCS) analysis

HCS is a tool for generating data on multiple parameters in single cell as well as in populations of cells, which is used to measure the toxicity or pro-apoptotic effect of compounds on cell [37, 38]. In this experiment, cells were incubated with FMG for 48 h, and stained with fluorescent dyes, including Hoechst33342, Annexin V-FITC and PI. Afterwards, plates were sealed and run immediately on the HCS Assay Scan VTI HCS Reader to acquire images. Images were analyzed with HCS software.

### Flow cytomery analysis of cell cycle and apoptosis

To determine cell cycle distribution and apoptosis, Propidium Iodide (PI) cell cycle kit and Alexa Fluor Annexin V and PI double staining (Annxin V/PI) kit were performed to detect by flow cytometer [33, 39]. Cells were seeded in 6-well plates at a density of 1 × 10^4^ cells/well and incubated for 24 h, and then exposed to FMG. After treatment for 12, 24 and 48 h respectively, the cells were stained with Annxin V/PI staining according to the manufacturer's instructions and suspended in a fluorochrome solution containing 5 μg/mL PI, 1 mg/mL sodium citrate, 0.1 mg/mL RNase and 1% Triton X-100 (Sigma-Aldrich, Sigma Chemical Co., St. Louis, MO, USA), and then analyzed with a flow cytometer (FACSCalibur, BD Instruments Inc., USA) and FlowJo 7.1.0 software (Tree Star, Ashland, OR, USA). All experiments were performed in triplicate, and for each measurement, at least 20,000 cells were counted.

### Wound healing assay

Cell migration was analyzed using the wound healing assay. At 90 % density in 6-well plates, a sterile 200 μL pipette tip was used to scratch a straight line through the cell layers and the culture medium was exposed to FMG. After treatment for 24 and 48 h respectively, cells were photographed 24 h, 48 h under an inverted phase contrast microscope (Olympus, Tokyo, Japan) after scratching. Assays were repeated three times for each clone.

### Cell invasion assay

The rate of cell invasion was monitored in RTCA DP with the xCELL Ligence system CIM-plates [40]. Briefly, the UC (Upper Chamber) of the CIM-plates was coated with Matrigel (BD Biosciences) for at 37°C, after incubated for 4 h, the cells were seeded in each well of the UC in serum-free media for 24 h, Then cells were treatment with FMG in each well of the UC and the fresh DMEM with 10% FBS was added to each well of the LC (Lower Chamber). The CIM-plates was left in an incubator for 1 h to allow cell attachment and the impedance value of each well was automatically monitored by the xCELL Ligence system for duration of 48 h and expressed as a CI value.

### Cytoskeletal rearrangement detection

Cytoskeletal rearrangement kit allows direct measurements of F-actin using a fixed end-point assay based on immunofluorescence detection using Thermo Scientific ArrayScan HCS Reader [41]. In this experiment, cells were incubated with FMG for 48 h, and stained with fluorescent dyes, including DAPI, Rhodamine phalloidin. Afterwards, plates were sealed and run immediately on the HCS Assay Scan VTI HCS Reader to acquire images. Images were analyzed with HCS software.

### Western blot assay

Cells were seeded in 6-well plates at a density of 1 × 10^4^ cells/well and incubated for 24 h, and then exposed to FMG for 6 h, 12 h, 24 h and 48 h, respectively. Proteins were resolved on SDS-PAGE and detected as described previously [42, 43]. The following antibodies were used in our studies: anti-p-PTEN, anti-t-PTEN, anti-p-AKT, anti-t-AKT, anti-cytc (cyto), anti-Bax, anti-Bcl-2, anti-p53 and β-actin.

### Simple western analysis

Simple western analyses were performed according to the ProteinSimple user manual [44, 45]. In brief, cells were seeded in 6-well plates at a density of 1 × 10^4^ cells/well and incubated for 24 h, and then exposed to FMG for 6 h, 12 h, 24 h respectively. And then cell lysate samples were mixed with a master mix to a final concentration of sample buffer, fluorescent molecular weight markers and dithiothreitol and then heated at 95 °C for 5 min. Next, the samples, blocking reagent, primary antibodies, chemiluminescent substrate, separation and stacking matrices were also dispensed to designated wells in a 384-well plate. After plate loading, the separation electrophoresis and immunodetection steps took place in the capillary system and were fully automated. The collected signal intensity was subsequently processed, converted to electropherogram, and integrated using Compass software (ProteinSimple).

### Immunofluorescence staining and high-content screening analysis

Immunofluorescence (IF) staining was performed using Thermo Scientific ArrayScan HCS Reader [46]. Briefly, cells were incubated with FMG for 24 h and standard IF procedures were performed. Cells were permeabilized with 0.5% Triton for 15 min and incubated with blocking solution for 30 min. The following primary antibody of p-PTEN was used for 1 h, and then Alexa Fluor-conjugated secondary antibody and DAPI were incubated for 1 h. Next, plates were sealed and run immediately on the HCS Assay Scan VTI HCS Reader to acquire images. Images were analyzed with HCS software.

### Protein expression and purification

The bacterial strains and plasmids used in this study were listed in Supplementary Table 8. The Human PTEN gene (1212bp, NCBI Reference Sequence NM_000314.4) was amplified by PCR using a plasmid as template which including PTEN coding region conserved in our lab and gene specific upstream primer 5’- CGGGATCCATGACAGCCATCATCAAA-3’ and downstream primer 5’- CCAAGCTTGACTTTTGTAATT-TGTGTA-3’ that included BamH I and Hind III restriction sites, respectively. The PCR products were subjected to the 1 % agarose gel electrophoresis, then purified using an agarose gel extraction kit and cloned into the pET28a vector. After DNA sequencing, the PTEN coding region was sub-cloned into pET28a expression vector and the plasmid construct was confirmed by restriction enzyme digestion. The blank empty plasmid was used as the negative control. The successfully constructed plasmid pET28a-PTEN was transformed into competent *E.coli* DH5α and *E.coli* BL21 and spread evenly on an 5 mL of Luria Bertani (LB) liquid medium containing Kanamycin (50 μg/mL), and cultured overnight at 37 °C with shaking at 210 rpm. Subsequently, the overnight culture was diluted into new 150 mL of fresh LB medium and incubated with *His*-PTEN fusion protein for 6 h in 37 °C at 210 rpm. Expression of *His*-PTEN was induced at different temperatures, isopropyl-β-D-thiogalactopyranoside (IPTG) concentration and induction time. To explore the optimum induction temperature for soluble *His*-PTEN fusion protein cells were cultured at 30, 37, 40 °C induced by 1mmol/L IPTG shaking at 210 rpm for 4 h. To determine the optimal concentration of IPTG for maximal yield of soluble protein, bacterium were induced with 0.1, 0.5, 1.0 mmol/L IPTG at 30 °C while shaking at 210 rpm for 4 h. Lastly, bacterium were induced with 1 mmol/L of IPTG for 2, 4, 6 h at 30 °C.

The bacterium were harvested by centrifugation at 10,000 g for 10 min at 4 °C and suspended in ice-cold PBS. Subsequently, sonication was carried out with 10 % power put out for 30 seconds by 3 times. To collect the inclusion bodied and soluble protein, the bacterium lysate was centrifuged at 10,000 g for 10 min at 4 °C and the supernatant was filtered with 0.22 μm filter before transferring into new tubes. Next, the ÄKTApurifier M1 protein chromatography purification system with HiTrap^TM^ Chelating HP chromatographic column (GE Healthcare, USA) were used to purify the *His*-PTEN protein and SDS-PAGE technique was performed to check the purification of the *His*-PTEN protein [47, 48].

### FMG and PTEN protein binding kinetic assay

Kinetics of FMG binding to PTEN protein was assessed using the Octet RED (ForteBio Inc., CA, USA) with Ni-NTA biosensor chips (ForteBio) as described [49–51]. Ni-NTA biosensors from ForteBio were pre-wetted for 5 min and used to capture *His*-PTEN onto the surface of the sensor for 600 sec. After reaching baseline for 60 sec, sensors were moved to association step in FMG solution for 600 sec and then dissociated for 600 sec. Curves were corrected by a double-referencing technique and the PTEN-FMG complex response, K_on_, K_dis_ and KD were calculated from global kinetic analysis. Data were collected by ForteBio data acquisition software and analyzed by ForteBio data analysis software.

### Phosphatase activity of PTEN protein detection

To detect the phosphatase activity of PTEN protein and PTEN+FMG complex, PTEN phosphatase activity assay kit was experimented (GENMED Scientifics Inc., USA). Briefly, *His*-PTEN fusion protein solution was desalted using the Zeba Desalt Spin Columns (Thermo Fisher Scientific Inc., Massachusetts, USA) and then quantified the protein concentration by BCA protein quantification assay kit (Thermo Fisher Scientific Inc., Massachusetts, USA). Subsequently, Samples were divided into PTEN control group and PTEN+FMG group (PTEN pre-interacted with FMG for 30 min) and tested by PTEN phosphatase activity assay kit according to the instructions.

### Statistical analysis

All experimental values were presented as means ± *SD*. Data were analyzed using SPSS 17.0 software. The significance of difference was determined by one-way *ANOVA* with the *LSD* tests, and Student's *t*-test was used for the two groups’ comparison as needed. Values of *P* < 0.05 were considered to be statistically significant.

## SUPPLEMENTARY MATERIALS FIGURES AND TABLES



## Abbreviations

FMG: The component formula of *Salvia miltiorrhiza* and *Panax* Ginseng, which is composed of Salvianolic acid A 5 μg/mL, 20(S)-Ginsenoside 5μg/mL and Ginseng polysaccharide 10 μg/mL
Sal A: Salvianolic acid A
Rh2: 20(S)-Ginsenoside
GPS: Ginseng polysaccharide
TCM: Traditional Chinese medicine
MTT: 3-(4,5-dimethylthiazol-2-yl)-2,5-diphenyltetrazolium bromide
RTCA: Real-time cell analysis
HCS: High content screening
NSCLC: Non-small cell lung cancer
PTEN: Phosphatase and tensin homolog
