# Supplementary material for: The component formula of Salvia miltiorrhiza and Panax ginseng induces apoptosis and inhibits cell invasion and migration through targeting PTEN in lung cancer cells
Source: Oncotarget. 2017 Sep 28;8(60):101599–613. doi: 10.18632/oncotarget.21354 (PMC5731899; doi:10.18632/oncotarget.21354)
Supplement: Supplementary file 1 [file oncotarget-08-101599-s001.pdf]

The component formula of *Salvia miltiorrhiza* and *Panax* Ginseng induces apoptosis and inhibits cell invasion and migration through targeting PTEN in lung cancer cells

SUPPLEMENTARY MATERIALS

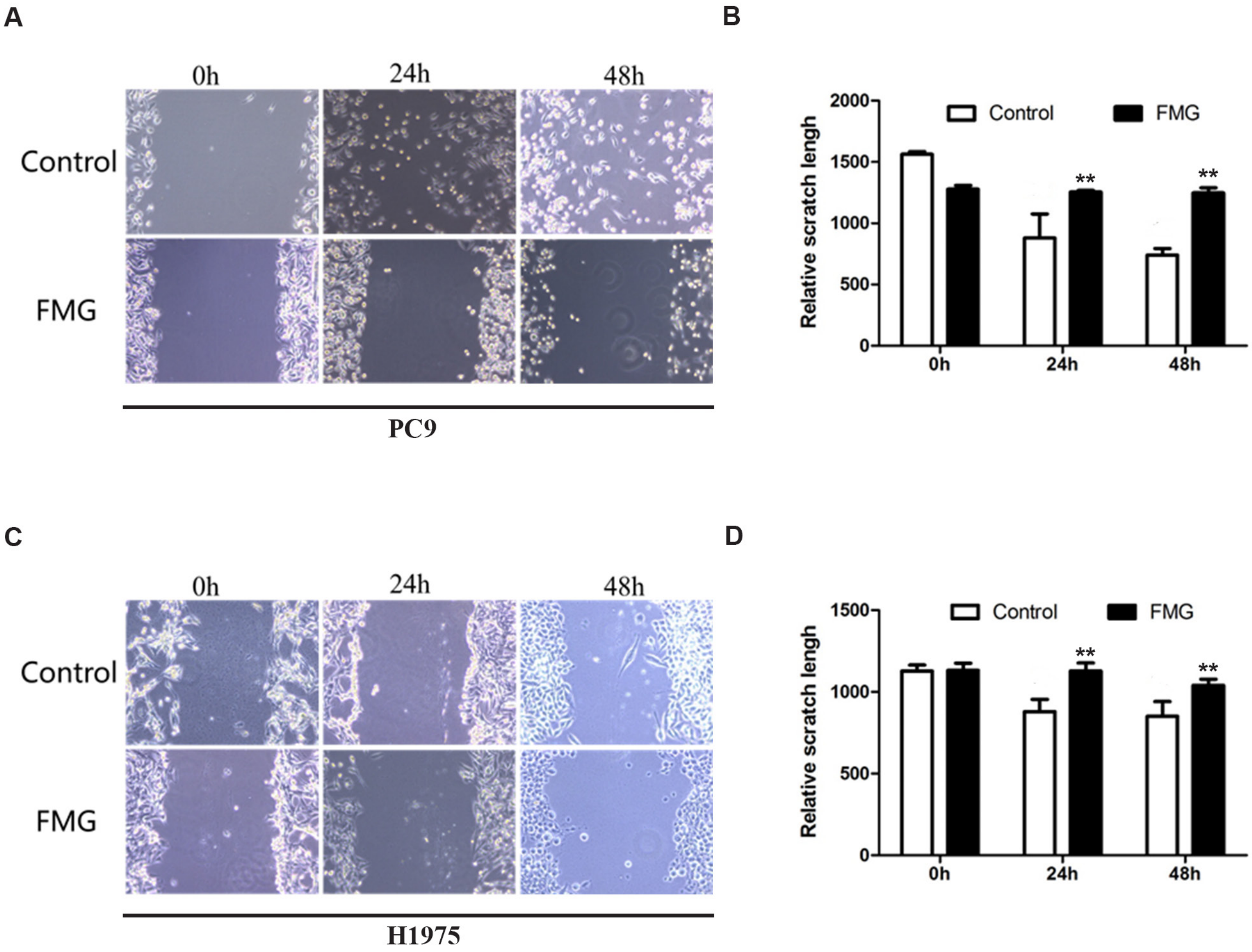

Supplementary Figure 1: The effects of FMG on cell migration in lung cancer cells. (A) Wound healing assays were used to assess the migration ability of PC9 cells after scratches. Cells were exposed to FMG for 24 and 48 h respectively, and then visualized by inverted phase contrast microscope. **(B)** Quantification of the relative scratch length in PC9 cells calculated by Imge J software. **(C)** Wound healing assays were used to assess the migration ability of H1975 cells after scratches. Cells were exposed to FMG for 24 and 48 h respectively, and then visualized by inverted phase contrast microscope. **(D)** Quantification of the relative scratch length in H1975 cells calculated by Image J software.

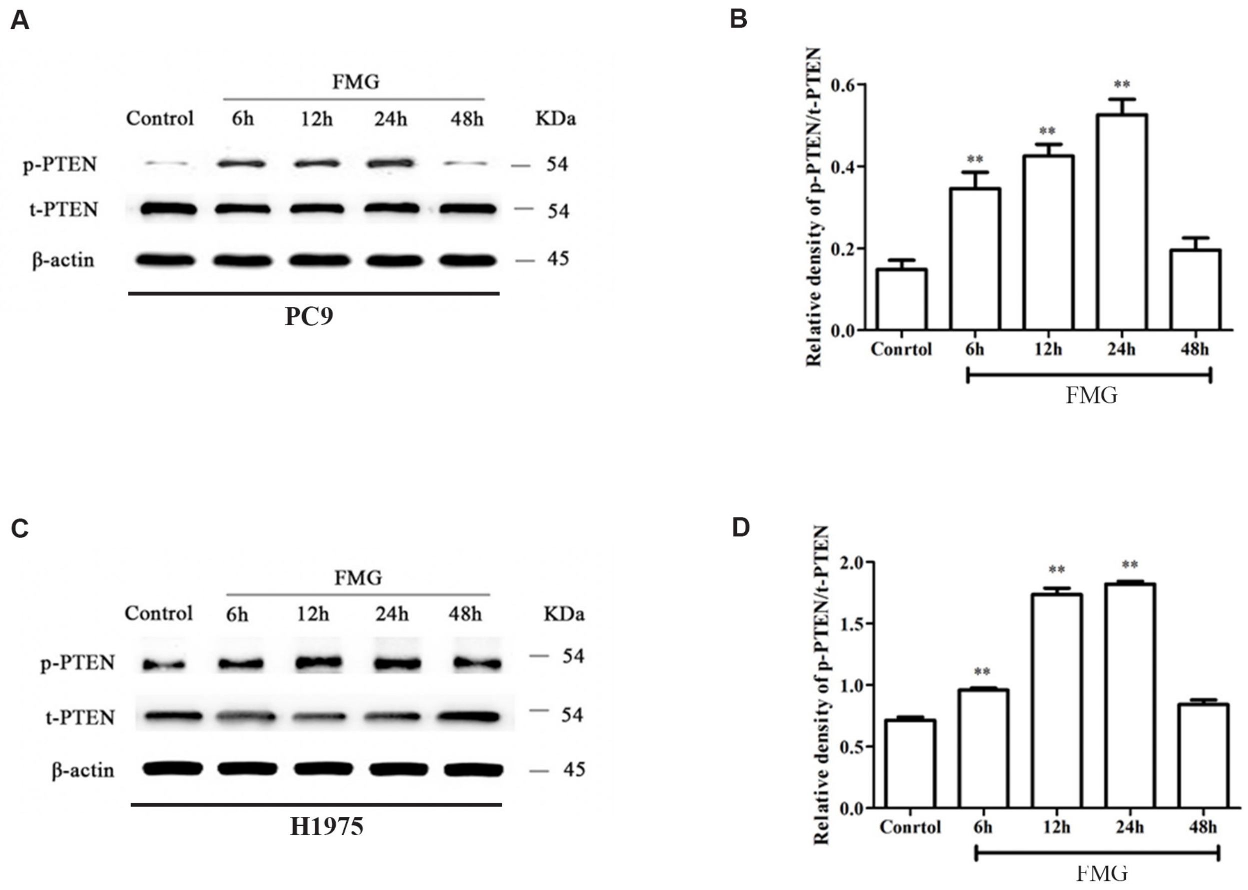

Supplementary Figure 2: The effects of FMG on the phosphorylation of PTEN in lung cancer cells. **(A)** PC9 cells were incubated with FMG for 6, 12, 24 and 48 h, respectively and then the p-PTEN, t-PTEN and β-actin were assessed via western blotting. **(B)** Quantification of p-PTEN/t-PTEN ratio folds of β-actin was calculated by Image J software in PC9 cells. Values expressed as mean ± SD from three independent experiments, $^{*}P < 0.05$, $^{**}P < 0.01$ vs. control group. **(C)** H1975 cells were incubated with FMG for 6, 12, 24 and 48 h, respectively and then the p-PTEN, t-PTEN and β-actin were assessed via western blotting. **(D)** Quantification of p-PTEN/t-PTEN ratio folds of β-actin was calculated by Image J software in H1975 cells. Values expressed as mean ± SD from three independent experiments, $^{*}P < 0.05$, $^{**}P < 0.01$ vs. control group.

Supplementary Table 1 : Optimizing the most effective component formula of Salviae Miltiorrhizae Radix et Rhizoma and Ginseng Radix et Rhizoma on cell proliferation of BEAS-2B and A549 cell line using the orthogonal design method ($\bar{x}$±s, n=3)

Groups	BEAS-2B		A549	
	A (OD)	IR (%)	A (OD)	IR (%)
Control	0.917±0.199		1.012±0.098	
1	0.193±0.003**	78.991±0.345	0.169±0.003**	83.335±0.286
2	0.181±0.000**	80.314±0.039	0.191±0.036**	81.096±3.525
3	0.805±0.007**	12.197±0.781	0.677±0.118*	33.156±11.612
4	0.185±0.002	79.816±0.167	0.167±0.002**	83.533 ±0.228
5	0.980±0.117	-6.889±12.789	0.847±0.015*	16.352±1.485
6	0.210±0.003**	77.086±0.359	0.165±0.003**	83.671±0.314
7	0.608±0.083	33.697±9.025	0.165±0.002**	83.740±0.181
8	0.194±0.007**	78.805±0.792	0.162±0.002**	83.970 ±0.227
9	0.184±0.005**	79.929±0.524	0.166±0.001**	83.582±0.120

Note:* $P < 0.05$, ** $P < 0.01$ *vs.* control group.

Supplementary Table 2: Univariate analysis of data generated from the L9 (3)4 orthogonal array

	Value		95% Confidence Interval	
	**Mean**	**Std. Error**	**Lower bound**	**Upper Bound**
A(Sal A)				
1	0.782	0.087	0.598	0.966
2	0.500	0.087	0.361	0.683
3	0.766	0.087	0.582	0.950
B(Rh2)				
1	0.769	0.087	0.585	0.952
2	0.517	0.087	0.333	0.701
3	0.761	0.087	0.577	0.945
C(Rg3)				
1	0.936	0.087	0.752	1.120
2	0.968	0.087	0.784	1.152
3	0.143	0.087	-0.041	0.327
D(GPS)				
1	0.509	0.087	0.325	0.926
2	0.772	0.087	0.588	0.956
3	0.767	0.087	0.583	0.950

Note: 1(10 μg/mL); 2(5 μg/mL); 3(0 μg/mL)
Sal A: Salvianolic acid A
Rh2: Ginsenoside Rh2
Rg3: Ginsenoside Rg3
GPS: Ginseng polysaccharide

Supplementary Table 3: Variance analysis of data generated from the L9 (3)4 orthogonal array

Source	Type III of Squares	df	Mean Square	F	*p*-value
Corrected Model	5.155[a]	8	0.644	9.355	<0.0001
Intercept	12.570	1	12.570	182.478	<0.0001
A (Sal A)	0.452	2	0.226	3.283	0.061
B (Rh2)	0.368	2	0.184	2.671	0.096
C (Rg3)	3.928	2	1.964	28.509	0.000
D (GPS)	0.407	2	0.204	2.957	0.078
Error	1.240	18	0.069		
Total	18.965	27			
Corrected Total	6.395	26			

Note: [a] R Squared=0.996 (Adjusted R Squared=0.880)
Sal A: Salvianolic acid A
Rh2: Ginsenoside Rh2
Rg3: Ginsenoside Rg3
GPS: Ginseng polysaccharide

Supplementary Table 4: The effects of FMG on cell proliferation in both BEAS-2B and lung cancer cells using MTT assay

Time	Group		OD	IR(%)
12h	BEAS-2B	Control	0.863±0.063	17.474±2.652
		FMG	0.712±0.229	
	H1975	Control	0.596±0.067	52.782±5.898
		FMG	0.282±0.035**	
	A549	Control	1.660±0.215	57.017±5.718
		FMG	0.714±0.095**	
	PC9	Control	0.771±0.140	43.561±2.336
		FMG	0.435±0.018**	
24h	BEAS-2B	Control	1.424±0.334	14.058±16.602
		FMG	1.224±0.236	
	H1975	Control	1.714±0.305	72.009±3.010
		FMG	0.480±0.052**	
	A549	Control	1.600±0.071	54.530±8.440
		FMG	0.728±0.135**	
	PC9	Control	1.937±0.520	32.370±4.529
		FMG	1.310±0.088**	
48h	BEAS-2B	Control	0.890±0.169	31.328±16.502
		FMG	0.147±0.012	
	H1975	Control	1.728±.0314	73.418±3.442
		FMG	0.459±0.059**	
	A549	Control	1.609±0.206	50.596±14.396
		FMG	0.795±0.232**	
	PC9	Control	2.027±0.338	40.186±5.472
		FMG	1.212±0.111**	

Supplementary Table 5: The affinity value of FMG and PTEN protein

No.	Group	Conc.(nM)	Response	Kon (1/Ms)	Kdis (1/s)	KD (M)	Full X^2	Full R^2
1	FMG	1.62×10^5	0.3537	9.70×10^3	3.14×10^{-1}	3.24×10^{-5}	11.133	0.906
2		8.09×10^4	0.2761					
3		4.04×10^4	0.1691					
4		2.02×10^4	0.106					
5		1.01×10^4	0.08					

Supplementary Table 6: Orthogonal design factors and levels of $L9(3)^4$

Levels	Factors			
	A (Sal A)	B (Rh_2)	C (Rg_3)	D (GPS)
1	1	1	1	1
2	2	2	2	2
3	3	3	3	3

Note: 1(10 µg/mL); 2(5 µg/mL); 3(0 µg/mL)
Sal A: Salvianolic acid A
Rh2: Ginsenoside Rh2
Rg3: Ginsenoside Rg3
GPS: Ginseng polysaccharide

Supplementary Table 7: The orthogonal design table of L9 $(3)^4$

Group	A (Sal A)	B (Rh2)	C (Rg3)	D (GPS)
1	1	1	1	1
2	1	2	2	2
3	1	3	3	3
4	2	1	2	3
5	2	2	3	1
6	2	3	1	2
7	3	1	3	2
8	3	2	1	3
9	3	3	2	1

Note: 1(10 µg/mL); 2(5 µg/mL); 3(0 µg/mL)
Sal A: Salvianolic acid A
Rh2: Ginsenoside Rh2
Rg3: Ginsenoside Rg3
GPS: Ginseng polysaccharide

Supplementary Table 8: Bacterial strains and plasmids

Strains and plasmids	Phenotypes
E.coli DH5α	F,ϕ80d/lacZ⊿M15,deoR,recA1,endA,hsdR17, phoA,supE44,λ-,thi-1,gyrA96,relA1
E.coli BL21	F-*dcm ompT hsdS*(rB-mB-)*gal*
$pET28a^+$	His-fusion expression vector, Kn^r
